# Role of Androgen Receptor CAG Repeat Polymorphism and X-Inactivation in the Manifestation of Recurrent Spontaneous Abortions in Indian Women

**DOI:** 10.1371/journal.pone.0017718

**Published:** 2011-03-14

**Authors:** Meka Aruna, Shilpi Dasgupta, Pisapati V. S. Sirisha, Sadaranga Andal Bhaskar, Surapaneni Tarakeswari, Lalji Singh, B. Mohan Reddy

**Affiliations:** 1 Molecular Anthropology Group, Biological Anthropology Unit, Indian Statistical Institute, Habsiguda, Hyderabad, India; 2 Lakshmi Fertility Clinic and Research Centre, Pogathota, Nellore, Andhra Pradesh, India; 3 Fernandez Hospital, Hyderabad, India; 4 Centre for Cellular and Molecular Biology, Hyderabad, India; University of Texas M. D. Anderson Cancer Center, United States of America

## Abstract

The aim of the present study was to investigate the role of CAG repeat polymorphism and X-chromosome Inactivation (XCI) pattern in Recurrent Spontaneous Abortions among Indian women which has not been hitherto explored. 117 RSA cases and 224 Controls were included in the study. Cases were recruited from two different hospitals - Lakshmi Fertility Clinic, Nellore and Fernandez Maternity Hospital, Hyderabad. Controls were roughly matched for age, ethnicity and socioeconomic status. The CAG repeats of the Androgen Receptor gene were genotyped using a PCR-based assay and were analysed using the GeneMapper software to determine the CAG repeat length. XCI analysis was also carried out to assess the inactivation percentages. RSA cases had a significantly greater frequency of allele sizes in the polymorphic range above 19 repeats (p = 0.006), which is the median value of the controls, and in the biallelic mean range above 21 repeats (p = 0.002). We found no evidence of abnormal incidence of skewed X-inactivation. We conclude that longer CAG repeat lengths are associated with increased odds for RSA with statistical power estimated to be ∼90%.

## Introduction

Recurrent Spontaneous Abortions (RSA) of unknown aetiology provide fundamental insights into the processes of embryogenesis and implantation and is also a frustrating and emotionally charged clinical problem. Spontaneous Abortion is one of the least understood pathological processes in spite of being one of the most common symptoms. Hormonal factors have been proposed to contribute to RSA [1], [2]. Hormonal aberrations may result from problems with certain endocrine glands, such as the pituitary, thyroid, adrenal and the ovaries. An increased miscarriage rate has been observed in women with polycystic ovarian disease, a disorder which is characterized by amenorrhoea, hirsutism, and raised androgen concentrations [1],[3]. The prevalence of polycystic ovarian syndrome (PCOS) in women with recurrent spontaneous abortion has been reported to be between 44% [1] and 82% [3]. Further, abnormalities in androgen secretion during the follicular phase, and hyperandrogenism have been associated with spontaneous abortions, suggesting an endocrine aetiology to this disorder [2]. At the molecular level, the effect of androgens is mediated through the activation of androgen receptor (AR). The human androgen receptor encoded in the X-chromosome contains a highly polymorphic CAG repeat sequence within exon 1. In vitro studies showed an inverse relation between number of CAG repeats in the AR gene and AR activity [4], and a number of clinical conditions, including infertility, have been attributed to the resulting variation in androgen activity has been cited by Mifsud et al. (2000) [5]. Few studies have found association of CAG repeats of the AR gene in PCOS [5]–[8] while many others did not [9]–[15]. But such investigations among RSA cases have not been hitherto conducted. Since AR gene lies on the X-chromosome, it becomes pertinent to assess the X chromosome Inactivation (XCI) pattern as well. There have been several studies where X-Inactivation patterns have been assessed in the RSA cases [16]–[22], particularly skewed XCI patterns, which suggest transmission of X-linked recessive lethal traits. However, the results are essentially confounding, while some studies reported association of skewed X-inactivation with RSA [16]–[18], others did not. Moreover, such studies have only been restricted to assess the skewed XCI pattern independent of the CAG repeat polymorphism among RSA women. Given the variation in the CAG repeat length of the androgen receptor (AR) gene and its inverse effect on the receptor activity, the alleles with longer CAG repeat length are expected to result in diminished androgen receptor activity, which might lead to a state of biochemical hyperandrogenism, i.e. increased serum levels of androgens as demonstrated in a couple of previous studies [5], [7]. Previous studies have reported hyperandrogenism to be associated with RSA where they have demonstrated elevated levels of androgens among RSA women in contrast to the controls [1], [2]. Therefore, the present study was undertaken to investigate the possible role of the CAG repeat polymorphism and the XCI pattern in the manifestation of RSA in the southern Indian women with the underlying hypothesis that longer CAG repeat length (resulting in hyperandrogenemia) would be associated with RSA.

## Materials and Methods

### Samples

Intravenous blood samples (∼5 ml) were collected from 117 RSA women with unknown aetiology, recruited from two different hospitals - 71 RSA cases from Lakshmi Fertility Clinic (LFC) in sub urban Nellore town and 46 RSA cases from Fernandez Maternity Hospital (FMH) in metropolitan Hyderabad. Blood samples from 224 control women, representing similar age, ethnicity and socioeconomic status as compared to cases, were drawn from general population of the respective areas (120 from Nellore and 104 from Hyderabad).

Recurrent Spontaneous Abortions (RSA) is defined as repeated occurrence of 3 or more miscarriages before 24th week of gestation [23]. The modern definition, however, is the spontaneous loss of 2 or more consecutive pregnancies before 20 weeks of gestation [24], [25]. Following the latest definition, women with ≥2 spontaneous abortions were included for the study. Other inclusion criteria considered for recruitment of cases were normal findings for: karyotype, anatomical features, tissue antibodies and auto antibodies, blood glucose levels and thyroid stimulating hormone concentrations and with no recent history of infectious aetiology. All the RSA women, with the mean age of 26 yrs (range 18–37 years of age) and number of miscarriages ranging between 2 and 9, had regular menstrual cycles and were healthy. Normal fertile controls with no history of abortions or treatment for fertility and with normal menstrual cycles every 25–32 days were recruited from the family planning centre of the Osmania hospital, Hyderabad and from the general population. The sampled subjects speak Telugu and the populations of these areas in Andhra Pradesh are observed to be genetically homogeneous [26].

### Ethics Statement

The samples were collected with written informed consent of all the subjects, cases as well as controls. This study has been approved by the Indian Statistical Institute Review Committee for Protection of Research Risks to Humans.

### DNA extraction, amplification and Genescan analysis

DNA was extracted from the peripheral blood samples of the patients and controls using the phenol-chloroform method [27]. The CAG repeats were genotyped using a PCR-based assay. Genomic DNA was amplified by PCR using fluorescently labelled primers that flank the CAG repeats [28]. The forward primer was labelled at the 5′end with the dye 6FAM. Amplification was carried out in a GeneAmp9700 thermal cycler (Applied Biosystems, Foster City, CA) as previously described [28]. The amplified products were separated on a denaturing polyacrylamide gel using an ABI 3730 genetic analyzer (Applied Biosystems, Foster City, CA). The fragment size was estimated by comparison with the internal size standard GS-LIZ500.

### X-Chromosome inactivation analysis

X inactivation analysis was carried out using the protocol given by Hickey et al. (2002)[7]. For the 341 heterozygous subjects, 100 ng of DNA were either digested with 1U of HpaII or incubated in digestion buffer alone at 37°C overnight, followed by incubation at 95°C for 5 min to denature the enzyme. Next, 1 µl of digested and mock-digested products were amplified using PCR primers given by Cram et al. (2000) [28] and peak areas of both the alleles were determined using an ABI 3730 genetic analyzer and Genemapper (version 3.7, Applied Biosystems). X inactivation (relative methylation of each allele) was quantified as previously described by Hickey et al. (2002) [7], comparing the ratio to which each allele contributed to the total peak area between digested and undigested samples.

The X-Inactivation percentages are expressed in terms of degree of inactivation of the longer allele. Nonrandom X inactivation is defined as more than 60% inactivation of either allele; skewed X inactivation is defined as more than 80% inactivation of either allele. Following X-Inactivation analysis, we calculated X-weighted biallelic means as per the protocol given by Hickey et al. (2002) [7], whereby each allele in a genotypic pair is multiplied by its percent activation, and the two adjusted repeat values are added together.

### Statistical Analysis

The number of different CAG repeats constitutes the different alleles in the sample. Frequency of each of these repeat lengths is considered to be the allele frequencies. χ2 analysis was done to test for the homogeneity of allele frequencies between the cases and controls. Logistic regression analysis was done and odds ratios obtained for perceived risk alleles (alleles which were observed to have relatively greater frequency among the RSA cases than the controls in the present study, i.e. CAG repeat size of >19 and CAG biallelic mean of ≥21) to check if significant association can be found with RSA. All the statistical analyses were performed with the help of SPSS statistical software (version 15.0, SPSS Inc, Chicago, IL, USA). For all tests, significance was set at 5%. Power Analysis of the logistic regression was carried out using G*Power (version 3.1.0, Germany).

## Results

### CAG allele distribution

**Figure 1:**
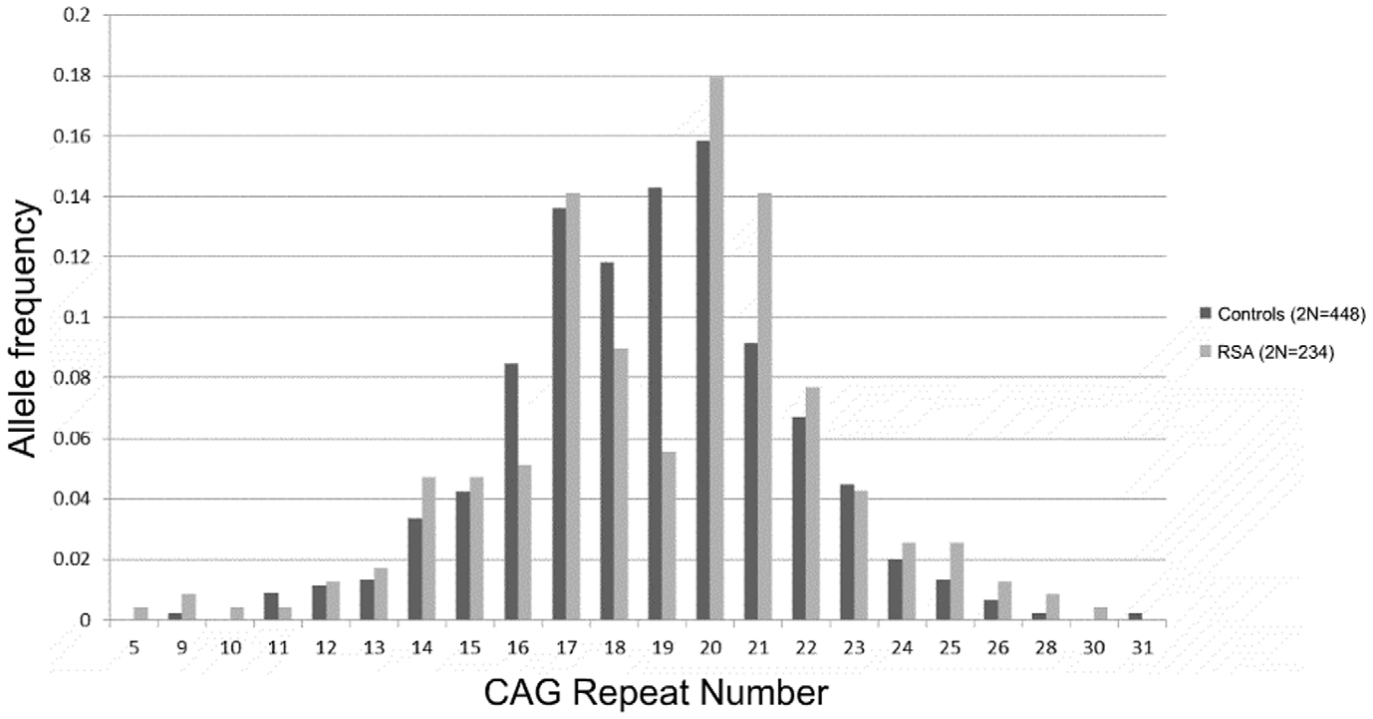
Distribution of CAG alleles in RSA cases and controls.

**Figure 2:**
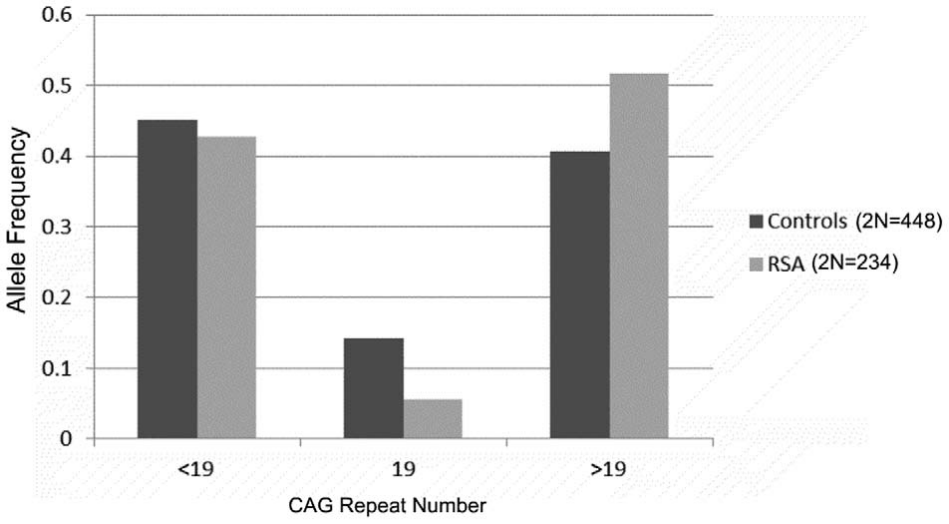
Frequency distribution of CAG alleles, grouped into 3 categories of <19, 19 and >19 CAG repeats, in RSA cases and controls.

**Figure 3:**
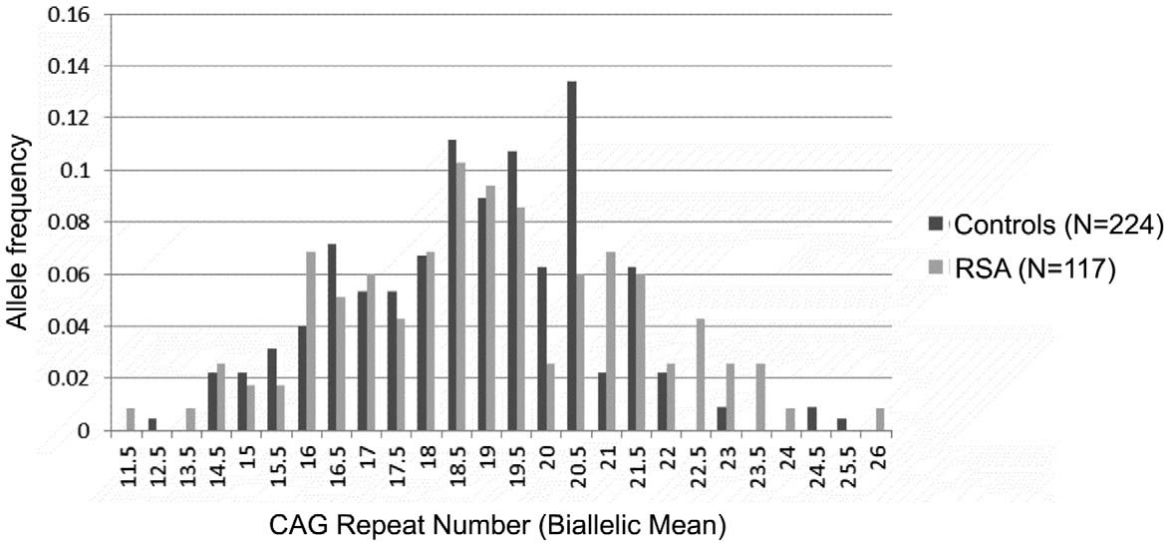
Distribution of CAG biallelic mean in RSA cases and controls.

In our cohort of 117 RSA women and 224 controls, the androgen receptor CAG repeats ranged from 5–30 and 9–31, respectively (Fig. 1). The mean number of CAG repeats for the cases (19.0±0.23) and controls (18.76±0.13) is similar. The median value of the CAG repeats is 20 for the cases and 19 for controls. For qualitative comparisons, the median value of 19 repeats observed for controls is considered, which bisects the entire polymorphic range providing three categories viz. <19, 19 and >19 repeats. The CAG allele distribution pattern of the RSA cases and controls is depicted in Figure 1. The allele distribution in the above three categories (Fig. 2) suggests significantly greater frequency of RSA cases in the >19 CAG repeat category (p = 0.0006). We also obtained a significant difference in the biallelic mean distribution pattern (Fig. 3) between the RSA cases and controls (p = 0.03) with the cases being more frequent in the upper polymorphic range. The frequency distribution of the RSA and control women in the three qualitative categories, bearing repeat values at the median, below and above the control median are presented in Table 1 for the total alleles (2N) and biallelic mean and in Table 2 for longer and shorter alleles separately. When the biallelic mean is categorised into <21 and ≥21 repeats, the RSA women were observed to be in a significantly higher frequency in the ≥21 repeat category (p = 0.002).

The case cohort was additionally analysed according to the number of abortions experienced by the subjects. The distribution of the CAG biallelic mean in different abortion categories is provided in Table S1. The case cohort was further divided into 2 groups: (i) cases with 2 abortions, and (ii) cases with 3 or more abortions. Allele frequency distribution was compared between these two groups and each of these groups with controls for biallelic mean and for both longer and shorter alleles separately. The χ2 analysis revealed significant heterogeneity in the biallelic mean distribution for each of the two abortion categories when compared with the controls (p = 0.013, p = 0.0007) as well as in the distribution of longer alleles between the RSA group with ≥3 abortions and the controls (p = 0.002) (Figure S1).

### Logistic Regression

**Table 1:** Distribution (%) of RSA cases and controls according to the qualitative categories of CAG repeats.

Repeat size	Total number of CAG alleles		CAG Biallelic Mean	
	Cases (2N = 234)	Controls (2N = 448)	Cases (N = 117)	Controls (N = 224)
<19	42.7	45.1	47.0	47.7
19*	5.5	14.2	8.9	9.4
>19	51.7	40.6	43.5	43.3

*Control median

**χ2 = 14.81, df = 2, p = 0.0006** χ2 = 0.29, df = 2, p = 0.985

**Table 2:** Distribution (%) of RSA cases and controls according to the qualitative categories of shorter and longer CAG repeats alleles (in an individual).

Repeat size	(i) Shorter CAG alleles		Repeat size	(ii) Longer CAG alleles	
	Cases (N = 117)	Controls (N = 224)		Cases (N = 117)	Controls (N = 224)
<17	38.4	36.6	<21	43.5	53.1
17*	16.2	15.2	21*	21.3	16.5
>17	45.2	48.2	>21	35.0	30.3

*Control Median

χ2 = 0.264, df = 2, p = 0.875 χ2 = 2.923, df = 2, p = 0.231

Given the significant heterogeneity in the biallelic mean frequency between the RSA cases and the controls, odds ratio was also computed to examine if there is any effect of repeat numbers in the upper polymorphic range of the allele spectrum in the manifestation of RSA. Odds were computed for the CAG repeats >19 VS≤19 for the total alleles and ≥21 VS<21 for the biallelic mean, considering the entire cohort and the two abortion categories separately with the controls (Table 3). Highly significant odds were obtained for each of the categories suggesting strong association of extreme CAG repeat lengths with RSA. Bonferroni correction for multiple testing was carried out for the logistic regression analysis. Except the RSA group with 2 abortions versus controls, all the other three categories show significant differences even after the correction.

**Table 3:** Results of logistic regression analysis for the extreme CAG repeat categories >19 VS≤19 and ≥21 VS<21 in total allele category (CAG RN) and for biallelic means (BAM), respectively.

	Cases		Controls		χ2	P-value	Odds ratio	95% CI for Odds ratio	
	(no)	%	(no)	%				Lower	Upper
a **CAG RN >19**	121	51.7	182	40.6	7.608	0.006**	1.56	1.13	2.15
a **BAM≥21**	31	26.4	29	12.9	9.058	0.002**	2.42	1.37	4.27
b **BAM≥21**	17	23.2	29	12.9	4.378	0.036*	2.04	1.04	3.98
c **BAM≥21**	14	31.8	29	12.9	9.058	0.003**	3.14	1.49	6.61

a Pooled RSA cases Vs Controls (For CAG RN, Cases: 2N = 234 Controls: 2N = 448; For BAM, Cases: N = 117 Controls: N = 224).

b RSA (2abortions) Vs Controls (Cases: N = 73 Controls: N = 224).

c RSA (≥3abortions) Vs Controls (Cases: N = 44 Controls: N = 224).

*not significant after Bonferroni correction;

**Significant after Bonferroni correction.

Regression analysis of each of the categories was subjected to post-hoc power analysis, which was carried out using G*Power software (version 3.1.0, Germany). Statistical power (1-β error probability) is computed as a function of significance level α, sample size, and population effect size (also defined by the odds ratio under the logistic regression criteria). Since the logistic regression results are being considered for power calculation, the conditional probability of the dependent variable Y and independent variable X under null hypothesis (H0) is also furnished as the input parameter denoted by Pr(Y = 1|X = 1)H0. Given our samples and setting α value at 0.05, we obtained very high power for our study (Table 4). We repeated the logistic regression and power analysis by selecting a 60% random subsample of the controls that results into similar sample size as that of the cases (results not presented), which yielded very similar results, qualitatively and quantitatively.

**Table 4:** Results of power analysis for different CAG allele categories used in the logistic regression alongwith the input parameters.

	Odds ratio	Pr(Y = 1|X = 1) H0	α err prob (P-value)	Total sample size	Binomial distribution parameter π	Power (1-β)
a **CAG RN >19**	1.56	0.34	0.05	682	0.44	0.878
a **BAM≥21**	2.42	0.34	0.05	341	0.18	0.997
b **BAM≥21**	2.04	0.24	0.05	297	0.15	0.679
c **BAM≥21**	3.14	0.16	0.05	268	0.16	0.916

a Pooled RSA cases Vs Controls (For CAG RN, Cases: 2N = 234 Controls: 2N = 448; For BAM, Cases: N = 117 Controls: N = 224).

b RSA (2abortions) Vs Controls (Cases: N = 73 Controls: N = 224).

c RSA (≥3abortions) Vs Controls (Cases: N = 44 Controls: N = 224).

### Internal Consistency

The RSA cases in our study were recruited from two hospitals: Lakshmi Fertility Clinic & Research Centre (LFC) and Fernandez Hospital (FMH). These two hospitals not only represent two different socioeconomic strata of the patients, but are also separated geographically by about 500 kms. The socio-economic status, which was assessed by taking into account the social hierarchy and occupational status reflecting the income patterns, is expected to affect the nutritional status of the individual and in turn the pregnancy outcome [29]–[32]. While the patients visiting FMH are from an urban background, and most of the patients fall into the middle or high income groups, the patients from LFC are from the semi urban and rural backgrounds and fall into middle and lower income categories. Therefore, in order to check for the internal consistency of our data, we analysed case samples from each of these hospitals separately with independent random subsamples of controls of comparable size to that of the respective case samples. Frequencies of the qualitative CAG repeat lengths (<19, 19 and >19 repeats) were compared in two groups: (i) RSA cases from FMH (N = 46) versus controls (N = 68) (subsample 1) and (ii) RSA cases from LFC (N = 71) versus controls (N = 87) (subsample 2). Concurrent to the pattern observed in the case of pooled sample, we found significantly greater frequency of RSA cases in the longer CAG repeat length category (>19 repeats) consistently in the two hospital specific analyses; while 57.6% of RSA cases had >19 CAG repeats as compared to 38.9% in controls in the case of FMH (χ2 = 9.88, df = 2, p = 0.007), 47.8% of RSA cases had >19 CAG repeats as compared to 40.2% in controls (χ2  = 6.19, df = 2, p = 0.045) in the case of LFC (Results not presented). The logistic regression analysis was further performed on each of the two hospital specific samples, which yielded significant odds ratios; CAG repeat length of >19 was associated with increased odds of RSA in each of the two samples (OR = 2.12, p = 0.006 and OR = 2.94, p = 0.015) albeit after Bonferroni correction, only the subsample 1 show significant association The detailed results for logistic regression analysis of these two categories are provided in Supplementary tables Table S2 and Table S3.

### X chromosome inactivation (XCI) analysis

**Figure 4:**
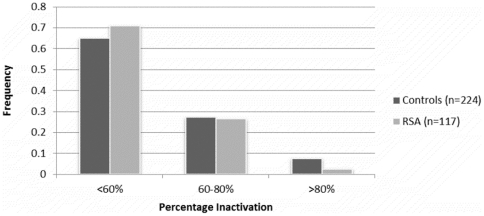
X-inactivation patterns in RSA cases and controls.

Analysis for X-chromosome inactivation revealed that majority of the cohort (71% of the RSA cases and 65% of the controls) follow random (<60%) X-inactivation pattern (Figure 4). We found no evidence of abnormal incidence of skewed X-inactivation (alleles ≥80% inactive) in either group (7.6% controls, 2.5% RSA), which is comparable to previously published studies [19]–[22]. X_weighted_biallelic mean, which was calculated by incorporating XCI percentages, also did not show any difference between cases and controls, hence no perceptible epigenetic influence can be deduced. The frequency distribution of the RSA and control women in the three qualitative categories, bearing repeat values at the median, below and above the control median is presented for X-weighted biallelic mean in Table 5. Allele frequency distribution was also compared between the two abortion groups and each of these groups with controls for X-weighted biallelic mean. However, none of these analyses yielded significant results to suggest epigenetic influence. Though we observed random XCI pattern for majority of the cases and controls, the X_weighted_biallelic distribution (Figure S2) depicts that the longer alleles are relatively more frequently active among the RSA cases compared to the controls.

**Table 5:** Distribution (%) of RSA cases and controls according to the qualitative categories of CAG X-weighted biallelic mean.

CAG X-weighted Biallelic Mean	Cases (N = 117)	Controls (N = 224)
<19	41.02	45.5
19	17.9	15.1
>19	41.02	39.2

χ2 = 0.779, df = 2, p = 0.677.

## Discussion

To the best of our knowledge, this is the first study that has examined the Androgen Receptor exon 1 CAG repeat variation in RSA. Our findings indicate that RSA women have a significantly greater frequency of longer AR CAG alleles (>19 repeats) and biallelic means (≥21 repeats) than fertile control women. The longer CAG repeat lengths were associated with increased odds of RSA. A number of earlier studies have focussed on the skewed X-Inactivation patterns among the RSA women yielding conflicting results, [16]–[22] only 3 of the 7 studies reported association [16]–[18]. In our case, the overall X-Inactivation pattern was not different between cases and controls, hence no perceptible epigenetic influence, which is in accordance to some of the previous studies where skewed XCI could not be associated with RSA [19]–[22]. It is pertinent to note that earlier investigations on the X-Inactivation patterns primarily addressed the issue of identification of the X-linked recessive lethal traits with the underlying genetic hypothesis that carriers of the recessive lethal traits manifest the molecular phenotype of non-random (skewed) X chromosome inactivation. In addition to this, it was also proposed that a subset of these lethal traits may cause an increased frequency of spontaneous abortions in carriers if the hemizygous trait produces a clinically detectable pregnancy [16].

Given the inverse relationship between the CAG repeat length of the androgen receptor (AR) gene and the receptor activity, the alleles with longer CAG repeat length are expected to show a state of biochemical hyperandrogenism Infact previous studies have reported hyperandrogenism to be associated with RSA where they have demonstrated elevated levels of androgens among RSA women in contrast to the controls [1], [2]. Therefore, we tried to examine the X-Inactivation patterns vis-a-vis the CAG repeat polymorphism of the AR gene. Although we did not observe any significant epigenetic influence, we could establish strong genetic association of CAG repeat length (>19 in case of total alleles and ≥21 in case of biallelic mean) with RSA yielding highly significant odds ratio with adequate statistical power (∼90%) conforming to our hypothesis. However, since the epigenetic influence was examined using peripheral blood samples in the present study and given that there are great variations in the degree of X-inactivation between ages and tissues [33], it thus becomes imperative to study the tissue specific XCI patterns in future (for eg. uterine cells, CVS) which might provide more unequivocal answers to the relative expression of CAG longer alleles and subsequent hyperandrogenic condition in RSA. Nevertheless, except in experimental situations including a couple of samples, it may not be feasible to obtain specific tissue samples especially in the Indian situation.

Individual analysis of the hospital-specific samples also yielded similar results to that of the pooled cohort, suggesting internal consistency in the data. Unfortunately, we could not obtain the serum androgen profiles of the cases in our study to verify if the biochemical hyperandrogenism is involved in the manifestation of RSA. Nevertheless, our study qualifies to provide the genetic evidence for possible biochemical hyperandrogenism [1], [2] as we could establish strong association of longer CAG repeat length with the RSA suggesting a possible endocrine aetiology of RSA. Further, while there was no significant difference in the CAG allele distribution patterns between the two abortion categories of our study, a highly significant difference was observed for CAG alleles with biallelic mean ≥21 repeats when these groups were separately compared with the controls. The higher abortion category exhibited a greater degree of difference with the controls which implies that longer CAG alleles may predispose women with a higher risk for spontaneous abortions.

In conclusion we may say that this maiden investigation of the CAG repeat polymorphism in RSA provides strong evidence of association of the longer CAG alleles with RSA, opening up the possibility of employing this as a meaningful prognostic genetic marker for identification of RSA cases. However, it is necessary to replicate and establish this association in other populations of the region before its prognostic value can be suggested with certainty. Further genetic and physiological (functional) studies are also required to ascertain the precise role of CAG polymorphism in the manifestation of this disorder, which may help in developing relevant endocrine therapeutic strategies for prevention of further miscarriages.

## Supporting Information

Table S1 Distribution of CAG biallelic mean (BAM) in different abortions categories among RSA women.
(DOC)

Table S2 Results of logistic regression analysis for the extreme CAG repeat categories >19 VS≤19 and ≥21 VS<21 in total allele category (CAG RN) and for biallelic means (BAM) in patients from Fernandez Maternity Hospital (FMH).
(DOC)

Table S3 Results of logistic regression analysis for the extreme CAG repeat categories >19 VS≤19 and ≥21 VS<21 in total allele category (CAG RN) and for biallelic means (BAM) in patients from Lakshmi Fertility Centre (LFC).
(DOC)

Figure S1 Distribution of CAG biallelic mean and longer alleles in RSA cases with ≥3 abortions and controls.
(TIF)

Figure S2 Distribution of CAG X_weighted_biallelic mean in RSA cases and controls.
(TIF)
