# Supplementary material for: Role of Androgen Receptor CAG Repeat Polymorphism and X-Inactivation in the Manifestation of Recurrent Spontaneous Abortions in Indian Women
Source: PLoS One. 2011 Mar 14;6(3):e17718. doi: 10.1371/journal.pone.0017718 (PMC3056719; doi:10.1371/journal.pone.0017718)
Supplement: Table S1 — Distribution of CAG biallelic mean (BAM) in different abortions categories among RSA women. (DOC) [file pone.0017718.s001.doc]

**Table S1:** Distribution of CAG biallelic mean (BAM) in different abortions categories among RSA women

|  | | Abortions | | | | | | Total |
| --- | --- | --- | --- | --- | --- | --- | --- | --- |
|  |  | 2 | 3 | 4 | 5 | 6 | 9 |  |
| BAM | 11.50 | 1 | 0 | 0 | 0 | 0 | 0 | 1 |
|  | 13.50 | 1 | 0 | 0 | 0 | 0 | 0 | 1 |
|  | 14.50 | 3 | 0 | 0 | 0 | 0 | 0 | 3 |
|  | 15.00 | 2 | 0 | 0 | 0 | 0 | 0 | 2 |
|  | 15.50 | 2 | 0 | 0 | 0 | 0 | 0 | 2 |
|  | 16.00 | 3 | 5 | 0 | 0 | 0 | 0 | 8 |
|  | 16.50 | 4 | 1 | 0 | 0 | 1 | 0 | 6 |
|  | 17.00 | 5 | 2 | 0 | 0 | 0 | 0 | 7 |
|  | 17.50 | 3 | 2 | 0 | 0 | 0 | 0 | 5 |
|  | 18.00 | 3 | 4 | 0 | 0 | 1 | 0 | 8 |
|  | 18.50 | 11 | 0 | 0 | 0 | 1 | 0 | 12 |
|  | 19.00 | 7 | 4 | 0 | 0 | 0 | 0 | 11 |
|  | 19.50 | 6 | 2 | 0 | 2 | 0 | 0 | 10 |
|  | 20.00 | 3 | 0 | 0 | 0 | 0 | 0 | 3 |
|  | 20.50 | 2 | 3 | 2 | 0 | 0 | 0 | 7 |
|  | 21.00 | 7 | 1 | 0 | 0 | 0 | 0 | 8 |
|  | 21.50 | 3 | 2 | 0 | 1 | 0 | 1 | 7 |
|  | 22.00 | 0 | 1 | 2 | 0 | 0 | 0 | 3 |
|  | 22.50 | 1 | 3 | 1 | 0 | 0 | 0 | 5 |
|  | 23.00 | 3 | 0 | 0 | 0 | 0 | 0 | 3 |
|  | 23.50 | 2 | 1 | 0 | 0 | 0 | 0 | 3 |
|  | 24.00 | 1 | 0 | 0 | 0 | 0 | 0 | 1 |
|  | 26.00 | 0 | 0 | 0 | 0 | 1 | 0 | 1 |
| Total | | 73 | 31 | 5 | 3 | 4 | 1 | 117 |
