# Supplementary material for: Role of Androgen Receptor CAG Repeat Polymorphism and X-Inactivation in the Manifestation of Recurrent Spontaneous Abortions in Indian Women
Source: PLoS One. 2011 Mar 14;6(3):e17718. doi: 10.1371/journal.pone.0017718 (PMC3056719; doi:10.1371/journal.pone.0017718)
Supplement: Table S2 — Results of logistic regression analysis for the extreme CAG repeat categories >19 VS≤19 and ≥21 VS<21 in total allele category (CAG RN) and for biallelic means (BAM) in patients from Fernandez Maternity Hospital (FMH). (DOC) [file pone.0017718.s002.doc]

**Table S2.** Results of logistic regression analysis for the extreme CAG repeat categories >19 VS ≤ 19 and ≥21 VS < 21 in total allele category (CAG RN) and for biallelic means (BAM) in patients from Fernandez Maternity Hospital (FMH)

| **LFC** | **Cases** | | |  | **Controls** | | | **χ2** | **P-value** | **Odds ratio** | **95% CI for Odds ratio** | |
| --- | --- | --- | --- | --- | --- | --- | --- | --- | --- | --- | --- | --- |
|  | **N** | **%** | |  | **N** | | **%** |  |  |  | **Lower** | **Upper** |
| **^a^CAG RN >19** | 53 | | 57.6 |  | 53 | 38.9 | | 7.56 | 0.006** | 2.12 | 1.24 | 3.64 |
| **^a^BAM** ≥ **21** | 13 | | 28.2 |  | 07 | 10.3 | | 5.71 | 0.016* | 3.43 | 1.24 | 9.44 |
| **^b^BAM** ≥ **21** | 05 | | 27.7 |  | 07 | 10.3 | | 3.35 | 0.067 | 3.35 | 0.92 | 12.22 |
| **^c^BAM** ≥**21** | 08 | | 28.5 |  | 07 | 10.3 | | 4.66 | 0.031* | 3.48 | 1.12 | 10.82 |

**^a^**Pooled RSA cases Vs Controls (For CAG RN, Cases: 2N=92 Controls: 2N=136; For BAM, Cases: N=46 Controls: N=68)

**^b^**RSA (2abortions) Vs Controls (Cases: N=18 Controls: N=68)

**^c^**RSA (≥3abortions) Vs Controls (Cases: N=28 Controls: N=68)

*not significant after Bonferroni correction; **significant after Bonferroni correction.
