# Supplementary material for: Role of Androgen Receptor CAG Repeat Polymorphism and X-Inactivation in the Manifestation of Recurrent Spontaneous Abortions in Indian Women
Source: PLoS One. 2011 Mar 14;6(3):e17718. doi: 10.1371/journal.pone.0017718 (PMC3056719; doi:10.1371/journal.pone.0017718)
Supplement: Table S3 — Results of logistic regression analysis for the extreme CAG repeat categories >19 VS≤19 and ≥21 VS<21 in total allele category (CAG RN) and for biallelic means (BAM) in patients from Lakshmi Fertility Centre (LFC). (DOC) [file pone.0017718.s003.doc]

**Table S3.** Results of logistic regression analysis for the extreme CAG repeat categories >19 VS ≤ 19 and ≥21 VS < 21 in total allele category (CAG RN) and for biallelic means (BAM) in patients from Lakshmi Fertility Centre (LFC)..

| **FMH** | **Cases** | | |  | **Controls** | | | **χ2** | **P-value** | **Odds ratio** | **95% CI for Odds ratio** | |
| --- | --- | --- | --- | --- | --- | --- | --- | --- | --- | --- | --- | --- |
|  | **N** | **%** | |  | **N** | | **%** |  |  |  | **Lower** | **Upper** |
| **^a^CAG RN >19** | 68 | | 47.8 |  | 70 | 40.2 | | 5.87 | 0.015* | 2.94 | 1.22 | 7.04 |
| **^a^BAM** ≥ **21** | 18 | | 25.3 |  | 09 | 10.3 | | 1.85 | 0.172 | 1.36 | 0.87 | 2.13 |
| **^b^BAM** ≥ **21** | 12 | | 21.8 |  | 09 | 10.3 | | 3.38 | 0.065 | 2.42 | 0.94 | 6.19 |
| **^c^BAM** ≥**21** | 06 | | 37.5 |  | 09 | 10.3 | | 6.95 | 0.008** | 5.20 | 1.52 | 17.70 |

**^a^**Pooled RSA cases Vs Controls (For CAG RN, Cases: 2N=142 Controls: 2N=174; For BAM, Cases: N=71 Controls: N=87)

**^b^**RSA (2abortions) Vs Controls (Cases: N=55 Controls: N=87)

**^c^**RSA (≥3abortions) Vs Controls (Cases: N=16 Controls: N=87)

*not significant after bonferroni correction; **Significant after Bonferroni correction.
