# Supplementary figures and images for: Role of Androgen Receptor CAG Repeat Polymorphism and X-Inactivation in the Manifestation of Recurrent Spontaneous Abortions in Indian Women
Source: PLoS One. 2011 Mar 14;6(3):e17718. doi: 10.1371/journal.pone.0017718 (PMC3056719; doi:10.1371/journal.pone.0017718)

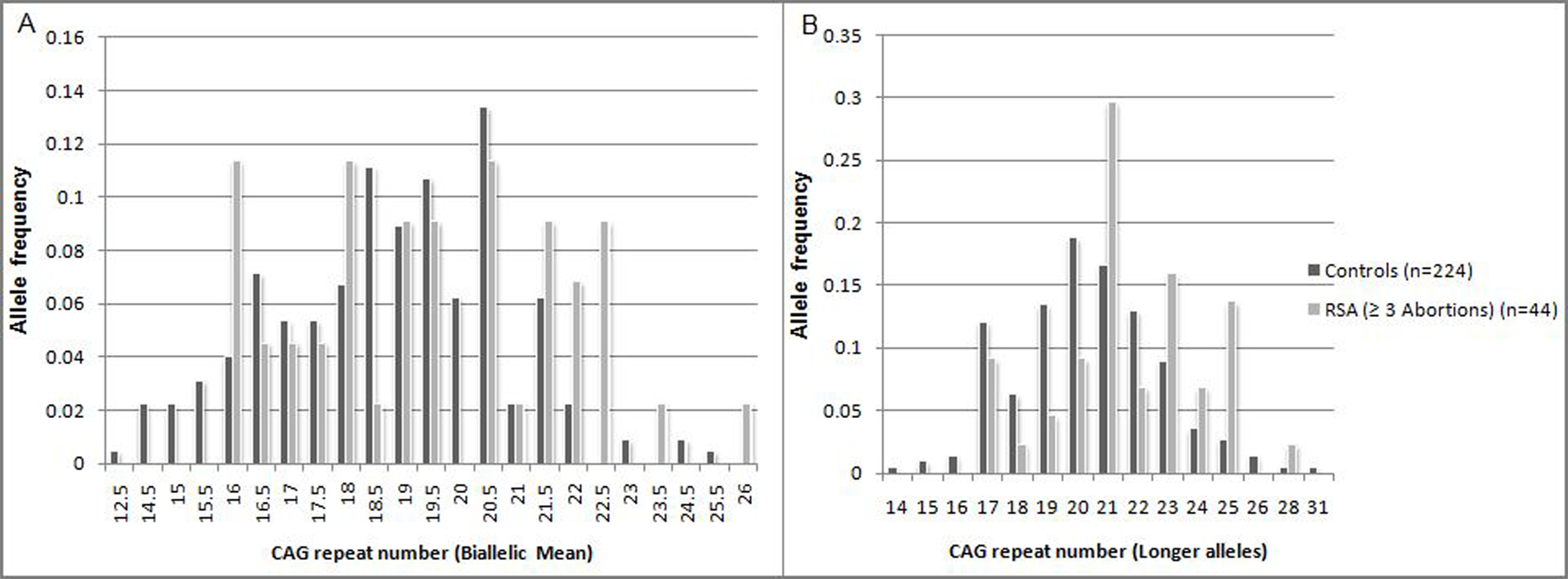

Supplement: Figure S1 — Distribution of CAG biallelic mean and longer alleles in RSA cases with ≥3 abortions and controls. (TIF) [file pone.0017718.s004.tif]

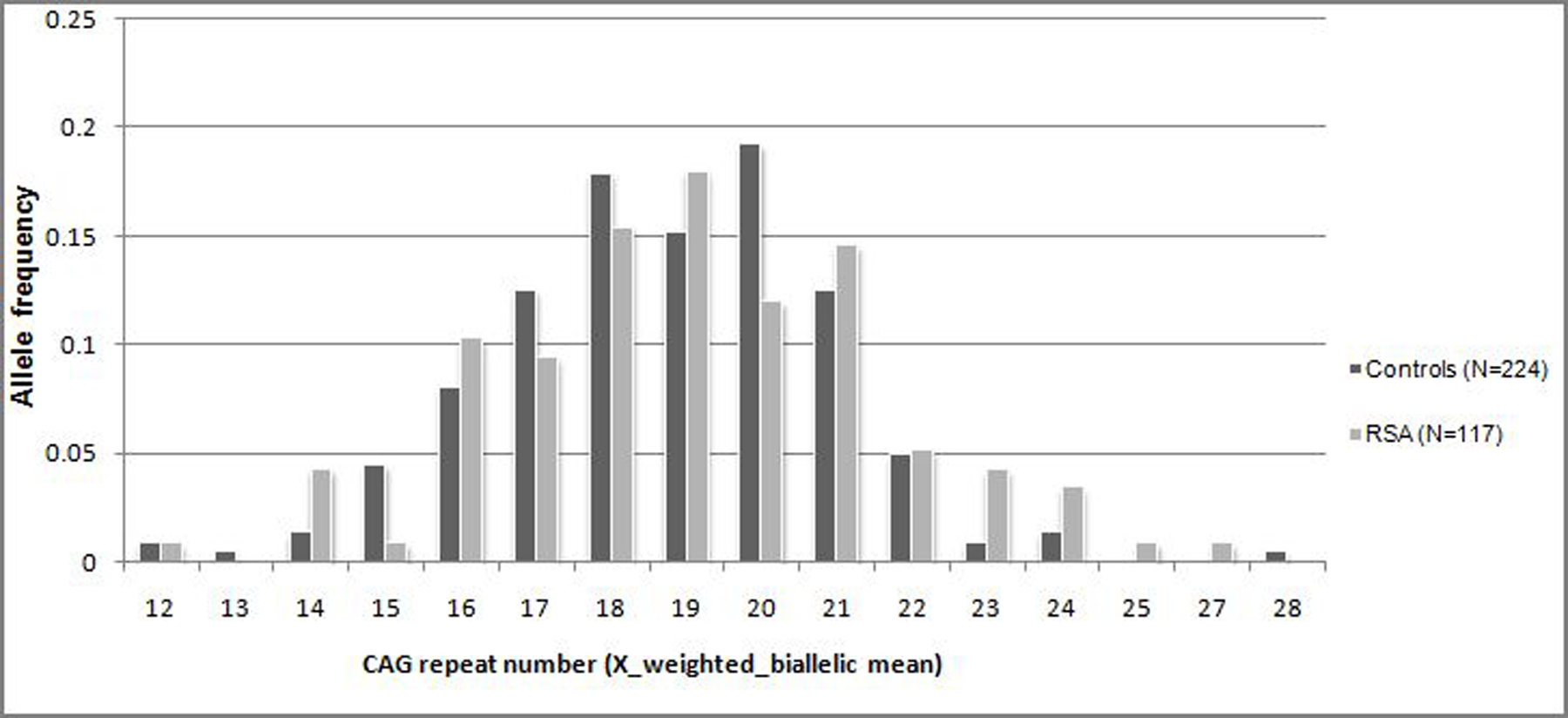

Supplement: Figure S2 — Distribution of CAG X_weighted_biallelic mean in RSA cases and controls. (TIF) [file pone.0017718.s005.tif]
